# A novel score based on serum apolipoprotein A-1 and C-reactive protein is a prognostic biomarker in hepatocellular carcinoma patients

**DOI:** 10.1186/s12885-018-5028-8

**Published:** 2018-11-28

**Authors:** Minjie Mao, Xueping Wang, Hui Sheng, Yijun Liu, Lin Zhang, Shuqin Dai, Pei-dong Chi

**Affiliations:** 1 Department of Laboratory Medicine, State Key Laboratory of Oncology in South China, Collaborative Innovation Center for Cancer Medicine, Sun Yat-sen University Cancer Center, Guangzhou, 510060 Guangdong People’s Republic of China; 2 Department of Experimental Research, State Key Laboratory of Oncology in South China, Collaborative Innovation Center for Cancer Medicine, Guangzhou, 510060 Guangdong People’s Republic of China

**Keywords:** HCC, Apolipoprotein A-1, C-reactive protein, Prognosis, Survival

## Abstract

**Background:**

The aim of this study was to propose a prognostic scoring system based on preoperative serum apolipoprotein A-1 and C-reactive protein (ApoA-1 and CRP, AC score) levels and to evaluate the prognostic value of these markers in patients with hepatocellular carcinoma (HCC).

**Methods:**

In all, 539 consecutive cases diagnosed with HCC from 2009 to 2012 at Sun Yat-sen University Cancer Center were analysed. The characteristics and levels of pretreatment lipids (ApoA-1, apolipoprotein B (Apo-B), high-density lipoprotein cholesterol (HDL-C), low-density lipoprotein cholesterol (LDL-C), total cholesterol (TC), triglycerides (TGs)) and CRP were reviewed and determined by univariate and multivariate Cox hazard models. Then, the AC score was proposed, which combines two independent risk factors (ApoA-1 and CRP)**.**

**Results:**

The optimal cut-off points in our study were determined according to established reference ranges. Patients with decreased ApoA-1 levels (< 1.090 g/L) and increased CRP levels (≥3.00 mg/L) exhibited a significantly poor overall survival (OS) and disease-free survival (DFS). The AC score was calculated as follows: patients with decreased ApoA-1 and elevated CRP were given a score of 3, patients with only one of these abnormalities were given a score of 2, and those with no abnormalities were given a score of 1. Patients with a higher AC score showed more progressive disease and a poorer prognosis. This was observed not only in the entire cohort (for OS, *P* < 0.001; for DFS, *P* < 0.001) but also in the subgroups stratified by pathological stage (stage I-II and stage III-IV). The discriminatory ability of the AC score in HCC was assessed according to the AUC values. The AUC value of the AC score (AUC: 0.676, 95% CI: 0.629–0.723, *P* < 0.001) was higher than that of AFP. In addition, the combination of the AFP and AC scores (AUC: 0.700, 95% CI: 0.655–0.745, *P* < 0.001) was superior to the AFP and AC scores alone.

**Conclusions:**

The AC score is a significant valuable predictor of OS and DFS and could more accurately differentiate the prognosis of HCC patients. As this study is a retrospective analysis, the value of the AC score should be validated in large prospective trials.

## Background

The incidence of primary liver cancer, which originates in the liver, has been increasing rapidly each year. Liver cancer accounted for 5.6% of all cancers according to data published in Globocan 2012 [1]. Among all types of primary liver cancers, hepatocellular carcinoma (HCC) is responsible for a major portion (accounts for approximately 80–90%), and more than half a million people have been diagnosed with HCC worldwide [2]. During the past several decades, various advances have been developed in the screening, diagnosis, and treatment of HCC. However, HCC is considered to be the third leading cause of all cancer-related deaths and is the fifth most common cancer worldwide [3]. The determination of alpha-fetoprotein (AFP) levels is the most commonly used screening test for HCC, but as many as 33.3% of HCC patients are AFP-negative. Therefore, a simple and instructive predictor is required to improve the prognoses of patients and provide better therapies.

Abnormal lipid metabolism has been reported to play an important role in tumour progression. It has also been demonstrated to be associated with increased risks of oesophageal squamous cell carcinoma [4], nasopharyngeal carcinoma [5, 6], and colorectal cancer [7], among other cancers. In particular, many studies have focused on the relationship between abnormal lipid levels and HCC. Jiang et al. [8] found that in HCC patients, decreased levels of CHO (cholesterol) and high-density lipoprotein cholesterol (HDL-C) might predict worse outcomes in terms of both DFS and OS. Ahaneku et al. [9] reported that a significant difference among HDL-fraction levels, including HDL-phospholipids (HDL-PL), HDL-cholesterol (HDL-C) and the ratio of HDL-C/HDL-PL, could be used to distinguish HCC patients and controls. Apolipoprotein A-1 (ApoA-1), the major protein form of HDL, is synthesized predominantly in the liver and small intestine. ApoA-1 has anti-inflammatory, anti-apoptotic, and antioxidant properties and inhibits the formation of tumour vessels [10]. Some studies have implied that ApoA-1 might play a significant role in tumorigenesis and cancer progression in HCC [11, 12]. However, limited data were available on the clinical significance of serum ApoA-1 levels in HCC. C-reactive protein (CRP), which indicates a systemic inflammatory response in cases of carcinoma, has been reported to affect carcinogenesis and tumour progression in cancers such as nasopharyngeal carcinoma [13]. Therefore, we hypothesized that in combination with CRP, which reflects inflammation and poor survival of cancer patients, ApoA-1 may be a potent prognostic indicator in HCC patients.

In this study, we established a simple and objective prognostic scoring system (the combined ApoA-1 and CRP score, termed the AC score) and elucidated the association of this score with the survival of HCC patients.

## Methods

### Patients

In all, the information system was accessed, and the data of 539 consecutive individuals with histologically confirmed HCC at Sun Yat-sen University Cancer Center from January 2009 to December 2012 were retrospectively reviewed. All the patients had undergone complete surgical resection. Patient characteristics, clinicopathological factors, and survival time were extracted from the Electronic Medical Record (EMR) system, whereas lipids, C-reactive protein (CRP) and alpha-fetoprotein (AFP) data were extracted from the Laboratory Information System (LIS). All these data are recorded in Table 1. Only the first record of hospitalization was retained, and the levels of lipids and AFP were investigated before treatment. However, cases with concomitant diseases that might have influenced the serum lipid levels (i.e., diabetes, hyperlipidaemia, or metabolic syndrome) were excluded. Patients with other types of tumours were also excluded. The tumour stage was evaluated according to the American Joint Committee on Cancer Staging system (AJCC, 2002; Greene). In all, 249 healthy participants (220 men and 29 women; ages 22–79 years, median 48 years) were examined by the Physical Examination Department of Sun Yat-sen University Cancer Center and were included in our study. Prior to the use of the serum, written informed consent was obtained from each of the patients and healthy participants. This study was approved by the ethics committees of Sun Yat-sen University Cancer Center (SYSUCC, Guangdong, China) and was conducted in accordance with the ethical standards of the World Medical Association Declaration of Helsinki.

**Table 1:** Characteristics and parameters of the 539 HCC patients

Characteristics	no. (%)	5-year OS (Months) Mean ± SD	*p*-Value	5-year DFS (Months) Mean ± SD	*p*-Value
Gender(n)					
Male	480(89.05)	30.82 ± 20.74	0.524	30.65 ± 20.71	0.566
Female	59(10.95)	29.71 ± 20.21		19.71 ± 20.21	
Age (years)					
≥ 53	275(51.02)	32.05 ± 20.57	0.167	31.75 ± 20.55	0.226
< 53	264(48.98)	29.30 ± 20.71		29.30 ± 20.71	
Stage(n)					
I and II	306(56.77)	38.16 ± 17.84	< 0.001	37.92 ± 17.87	< 0.001
III and IV	233(43.23)	20.90 ± 20.06		20.87 ± 20.07	
Tumor size(cm)					
≥ 5	220(40.82)	20.17 ± 19.68	< 0.001	20.14 ± 19.69	< 0.001
< 5	319(59.18)	37.96 ± 18.06		37.73 ± 18.09	
Tumor number					
Single	335(62.15)	33.71 ± 20.02	0.001	33.55 ± 20.04	0.001
Multiple	192(35.62)	26.74 ± 20.75		26.60 ± 20.66	
Node stage					
N0	502(93.14)	31.35 ± 20.34	0.016	31.19 ± 20.32	0.017
N1–2	37(6.86)	21.92 ± 23.30		21.92 ± 23.30	
Distant metastases					
Yes	31(5.75)	24.52 ± 23.08	0.102	24.52 ± 23.08	0.108
No	508(94.25)	31.08 ± 20.48		30.92 ± 20.45	
BMI (kg/m^2^)					
≥ 24.0	174(32.28)	32.70 ± 20.70	0.234	32.44 ± 20.58	0.292
18.5–23.9	319(59.18)	29.94 ± 20.74		29.83 ± 20.77	
< 18.5	45(8.35)	28.80 ± 19.90		28.80 ± 19.90	
Alcohol behavior					
Previous/Current	200(37.11)	30.02 ± 21.18	0.683	29.66 ± 21.08	0.535
Never	339(62.89)	31.10 ± 20.38		21.08 ± 20.40	
Family history of cancer					
Yes	138(25.60)	32.21 ± 20.52	0.362	32.21 ± 20.52	0.303
No	401(74.40)	30.18 ± 20.72		29.98 ± 20.68	
HBs Ag					
Negative	61 (11.32)	30.23 ± 19.87	< 0.001	30.22 ± 19.87	< 0.001
Positive	362(67.16)	39.98 ± 18.05		39.77 ± 18.05	
HBe Ag					
Negative	361(66.98)	31.75 ± 20.65	0.283	31.56 ± 20.64	0.317
Positive	62(11.50)	28.44 ± 21.28		28.44 ± 21.28	
AFP (ng/mL)					
≥ 400	195(36.18)	24.35 ± 20.46	< 0.001	24.19 ± 20.40	< 0.001
< 400	323(59.93)	35.85 ± 19.18		35.70 ± 19.19	
ALB					
≥ 41.35	271(50.28%)	35.40 ± 19.56	< 0.001	35.20 ± 19.58	< 0.001
< 41.35	268(49.72%)	25.95 ± 20.71		25.85 ± 20.66	
TBIL					
≥ 15.95	209(38.78%)	26.08 ± 21.20	< 0.001	26.02 ± 21.24	< 0.001
< 15.95	330(61.22%)	33.63 ± 19.80		33.42 ± 19.78	
HDL(mmol/L)					
≥ 1.16	253(46.94)	34.25 ± 19.56	< 0.001	34.00 ± 19.61	< 0.001
< 1.16	286(53.06)	27.56 ± 21.14		27.50 ± 21.08	
LDL(mmol/L)					
≥ 3.1	276(51.21)	30.72 ± 20.87	0.924	30.64 ± 20.82	0.870
< 3.1	263(48.79)	30.68 ± 20.49		30.46 ± 20.49	
ApoA-1(g/L)					
≥ 1.09	279(51.76)	36.22 ± 19.36	< 0.001	35.99 ± 19.42	< 0.001
< 1.09	260(48.24)	24.78 ± 20.42		24.71 ± 20.35	
ApoB(g/L)					
≥ 1.1	97(18.00)	29.91 ± 21.97	0.717	29.87 ± 21.96	0.761
< 1.1	442(82.00)	30.88 ± 20.39		30.70 ± 20.37	
CRP(mg/L)					
≥ 3.0	290(53.80)	22.63 ± 20.25	< 0.001	22.57 ± 20.29	< 0.001
< 3.0	249(46.20)	40.10 ± 16.85		39.85 ± 16.84	

Abbreviations:*OS* overall survival, *DFS* disease-free survival, *AFP* alpha fetoprotein, *TC* total cholesterol, *TG* triglycerides, *HDL-C* high-density lipoprotein cholesterol, *LDL-C* low-density lipoprotein cholesterol, *ApoA-1* apolipoprotein A-1, *ApoB* apolipoprotein B, *CRP* C-reactive protein

### Follow-up

All HCC patients were advised to receive regular follow-up after completion of primary treatment according to the clinical guidelines. Patients were generally followed-up every 3 months by the outpatient service with ultrasound in the first 2 years, but patients without evidence of recurrence were followed-up annually for the next 3 to 5 years. For those who did not visit our hospital as scheduled, telephone follow-ups were conducted to obtain treatment information and living status (performed by The Medical Information Unit in our Cancer Center). The last follow-up was in December 2016. The outcomes of our study were overall survival (OS) and disease-free survival (DFS). OS was defined as the time from the diagnosis of HCC to the date of the last follow-up or death. DFS was calculated as the time between the first diagnosis and the date of disease recurrence.

### Laboratory measurements

Patients underwent routine serological and biochemical tests at the first visit to our hospital. Serum samples were collected between 7 and 8 AM, clotted at room temperature, and centrifuged at 3500 r/min for 8 min. A Hitachi 7600 automatic biochemical analyser (Hitachi High Technologies, Tokyo, Japan) was used to measure the level of lipids. TC was tested by the CHOD-PAP method, while TGs were measured by the GPO-PAP method. HDL-C and LDL-C were measured by the selective elimination method (direct method) and the selective protection method, respectively. ApoA-1and Apo-B were detected by the immunoturbidimetry method. All reagents for the detection of lipids were provided by Wako Pure Chemical Industries, Japan. The level of AFP was tested using a Roche E-170 automatic electrochemistry analyser (Basel, Switzerland). The reagent for AFP detection was provided by Roche.

### Statistical analysis

Statistical analysis was performed using SPSS 16.0 for Windows software (IBM, Chicago, IL, USA). All the optimal cut-off points in our study were determined according to established reference ranges. Data were expressed as the mean and standard deviation (mean ± SD). To investigate lipid abnormalities in HCC patients, the nonparametric test was used to compare the levels of lipids and CRP. The differences between HCC patients and healthy donors were compared by the nonparametric test using the Mann-Whitney U test and were plotted by GraphPad Prism 5. Univariate and multivariate regression analyses were used to analyse the associations of lipids with cancer. The Kaplan-Meier method and the log-rank test were used to plot the survival curves, and the correlation between the AC score and clinical characteristics was assessed using the *Χ*^2^ test. The discriminatory ability of a factor to predict survival was assessed using the area under the curve (AUC). A two-tailed *P* value < 0.05 was considered statistically significant.

## Results

### The relationships between clinicopathologic characteristics and survival time

The clinical characteristics of the 539 HCC patients are shown in Table 1. Of the patients, 480 were men (89.05%) and 59 were women (10.95%), and the median age was 53 years (range: 19 to 88 years). In the entire cohort, 306 (56.77%) patients were diagnosed with pathological stage I or II disease, while 233 (43.23%) patients were diagnosed with pathological stage III or IV disease. Patients with advanced stage disease (III and IV) have a shorter OS and DFS than those with early-stage disease (OS: 20.90 ± 20.06 vs. 38.16 ± 17.84, *P* < 0.001; DFS: 20.87 ± 20.07 vs. 37.92 ± 17.87, *P* < 0.001). Patients with HBsAg positivity demonstrated better OS and DFS (OS: 39.98 ± 18.05 vs. 30.23 ± 19.87, *P* < 0.001; DFS: 39.77 ± 18.05 vs. 30.22 ± 19.87, *P* < 0.001). The serum biomarkers AFP, HDL, ApoA-1 and CRP also influenced the OS and DFS of HCC patients.

### Pretreatment lipids and CRP levels were compared between HCC patients and healthy controls

**Fig. 1:**
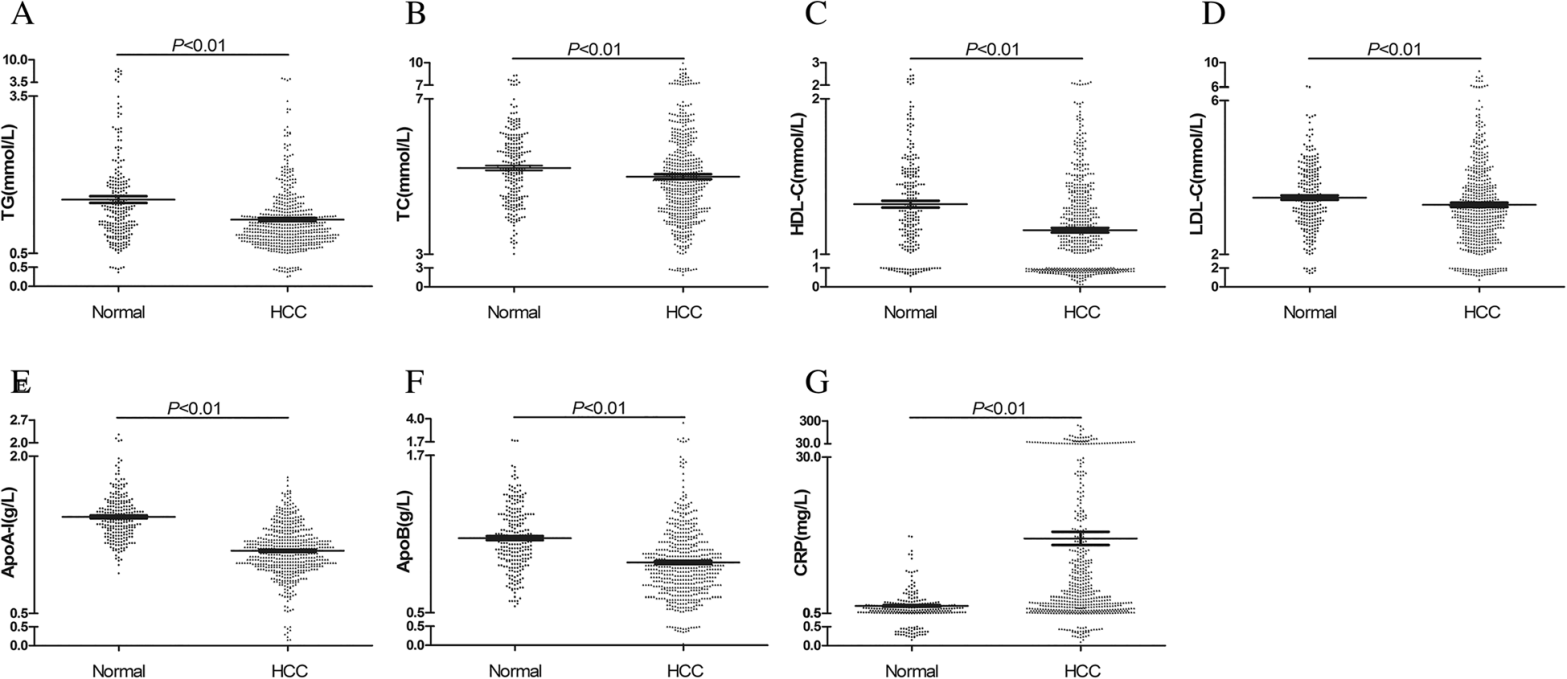
Comparison of pretreatment serum lipids and CRP in HCC patients and healthy controls. (**a**) The differences in TG level; (**b**) The differences in TC level; (**c**) The differences in HDL-C level; (**d**) The differences in LDL-C level; (**e**) The differences in ApoA-1 level; (**f**) The differences in ApoB level; (**g**) The differences in CRP level

To investigate lipid abnormalities in HCC patients, the nonparametric test was used to compare the levels of lipids and CRP. The levels of TGs (1.15 ± 0.71 mmol/L), TC (5.00 ± 1.48 mmol/L), HDL-C (1.16 ± 1.03 mmol/L), LDL-C (3.19 ± 1.27 mmol/L), ApoA-1 (1.10 ± 0.24 g/L), and Apo-B (0.88 ± 0.29 g/L) in HCC patients were significantly lower than those in the control group, which was age- and sex-matched (TG: 1.53 ± 0.71 mmol/L, TC: 5.22 ± 0.97 mmol/L, HDL-C: 1.32 ± 0.35 mmol/L, LDL-C: 3.48 ± 0.88 mmol/L, ApoA-1: 1.42 ± 0.22 g/L, Apo-B: 1.07 ± 0.25 g/L). In addition, the CRP level (14.61 ± 28.86 mg/L) was higher in HCC patients compared with healthy controls (1.93 ± 2.34 mg/L) (Fig. 1).

### Prognostic significance of the independent predictors in HCC

All the available information, including the clinicopathologic characteristics and biomarkers, were included in the univariate and multivariate analyses to analyse the association between serum HBsAg and cancers. In the univariate analyses, a significant correlation was observed among TNM stage (*P* < 0.001 vs. *P* < 0.001), tumour number (*P* < 0.001 vs. *P* < 0.001), size (*P* < 0.001 vs. *P* < 0.001), node stage (*P* < 0.001 vs. *P* < 0.001), distant metastases (*P* = 0.008 vs. *P* = 0.008), AFP (*P* < 0.001 vs. *P* < 0.001), HDL (*P* = 0.011 vs. *P* = 0.025), ApoA-1 (*P* < 0.001 vs. *P* < 0.001), CRP (*P* < 0.001 vs. *P* < 0.001), and OS and DFS. Multivariate analyses were then performed to identify factors that were distinguished in the univariate analyses. The results showed that TNM stage (HR: 3.068, 95% CI: 2.263–4.160, *P* < 0.001), AFP (HR: 1.710, 95% CI: 1.292–2.264, *P* < 0.001), ApoA-1 (HR: 0.588, 95% CI: 0.440–0.786, *P* < 0.001) and CRP (HR: 2.056, 95% CI: 1.491–2.834, *P* < 0.001) were identified as significantly independent predictors of OS in HCC patients (Table 2). Moreover, TNM stage (HR: 3.068, 95% CI: 2.23–4.160, *P* < 0.001), AFP (HR: 1.710, 95% CI: 1.292–2.264, *P* < 0.001), ApoA-1 (HR: 0.588, 95% CI: 0.440–0.786, *P* < 0.001) and CRP (HR: 2.056, 95% CI: 1.491–2.834, *P* < 0.001) were independent prognostic indicators of DFS (Table 3).

**Table 2:** Univariate and multivariate cox hazards analysis for overall survival in 539 patients with HCC

Variables	Univariate analysis		Multivariate analysis		
	*Χ* ^2^	*p*-Value	HR	95%CI	*p*-Value
Gender					
Male vs. Female	0.035	0.853			
Age (years)					
< 53 vs. ≥ 53	0.481	0.488			
TNM stage					
III-IV vs. I-II	122.544	< 0.001	3.068	2.263–4.160	< 0.001
Tumor size(cm)					
< 5 vs. ≥ 5	122.424	< 0.001			
Tumor number					
Single vs. Multiple	20.516	< 0.001			
Node stage					
N0 vs. N1–2	15.215	< 0.001			
Distant metastases					
Yes vs. No	7.140	0.008			
BMI (kg/m^2^)					
≥ 24.0 vs. 18.5–23.9 vs. < 18.5	4.454	0.108			
Alcohol behavior					
Previous/Current vs. Current	0.193	0.908			
Family history of cancer					
Yes vs. No	3.851	0.146			
HBs Ag					
Positive vs. Negative	0.749	0.384			
HBe Ag					
Positive vs. Negative	2.015	0.156			
AFP (ng/mL)					
< 400 vs. ≥400	27.697	< 0.001	1.710	1.292–2.264	< 0.001
TC(mmol/L)					
< 4.625 vs. ≥4.625	0.241	0.624			
TG(mmol/L)					
< 0.615 vs. ≥0.615	0.154	0.695			
HDL(mmol/L)					
< 1.085 vs. ≥1.085	6.534	0.011			
LDL(mmol/L)					
< 2.695 vs. ≥2.695	20.524	0.469			
ApoA-1(g/L)					
< 1.075 vs. ≥1.075	34.756	< 0.001	0.588	0.440–0.786	< 0.001
ApoB(g/L)					
< 0.635 vs. ≥0.635	0.854	0.356			
CRP(mg/L)					
< 6.805 vs. ≥ 6.805	73.715	< 0.001	2.056	1.491–2.834	< 0.001

Abbreviations: *HR* hazard ratio, *95% CI* 95% confidence interval, *BMI* body mass index, *AFP* alpha fetoprotein, *TC* total cholesterol, *TG* triglycerides, *HDL-C* high-density lipoprotein cholesterol, *LDL-C* low-density lipoprotein cholesterol, *ApoA-1* apolipoprotein A-1, *ApoB* apolipoprotein B, *CRP* C-reactive protein

**Table 3:** Univariate and multivariate cox hazards analysis for Disease-free survival in 539 patients with HCC

Variables	Univariate analysis		Multivariate analysis		
	*Χ* ^2^	*p*-Value	HR	95%CI	*p*-Value
Gender					
Male vs. Female	0.030	0.861			
Age (years)					
< 53 vs. ≥ 53	0.423	0.515			
TNM stage					
III-IV vs. I-II	121.676	< 0.001	3.068	2.263–4.160	< 0.001
Tumor size(cm)					
< 5 vs. ≥ 5	121.603	< 0.001			
Tumor number					
Single vs. Multiple	20.408	< 0.001			
Node stage					
N0 vs. N1–2	15.031	< 0.001			
Distant metastases					
Yes vs. No	7.069	0.008			
BMI (kg/m^2^)					
≥ 24.0 vs. 18.5–23.9 vs. < 18.5	4.431	0.109			
Alcohol behavior					
Previous/Current vs. Current	0.178	0.915			
Family history of cancer					
Yes vs. No	3.929	0.140			
HBs Ag					
Positive vs. Negative	0.778	0.378			
HBe Ag					
Positive vs. Negative	1.937	0.164			
AFP (ng/mL)					
< 400 vs. ≥400	28.609	< 0.001	1.710	1.292–2.264	< 0.001
TC(mmol/L)					
< 5.69 vs. ≥5.69	0.182	0.670			
TG(mmol/L)					
< 1.7 vs. ≥1.7	0.626	0.429			
HDL(mmol/L)					
< 1.16 vs. ≥1.16	5.056	0.025			
LDL(mmol/L)					
< 3.1 vs. ≥3.1	0.292	0.589			
ApoA1(g/L)					
< 1.20 vs. ≥1.20	30.090	< 0.001	0.588	0.440–0.786	< 0.001
ApoB(g/L)					
< 1.10 vs. ≥1.10	0.675	0.411			
CRP(mg/L)					
< 3.0 vs. ≥ 3.0	64.842	< 0.001	2.056	1.491–2.834	< 0.001

Abbreviations:*HR* hazard ratio, *95% CI* 95% confidence interval, *BMI* body mass index, *AFP* alpha fetoprotein, *TC* total cholesterol, *TG* triglycerides, *HDL-C* high-density lipoprotein cholesterol, *LDL-C* low-density lipoprotein cholesterol, *ApoA-1* apolipoprotein A-1, *ApoB* apolipoprotein B; *CRP* C-reactive protein

**Fig. 2:**
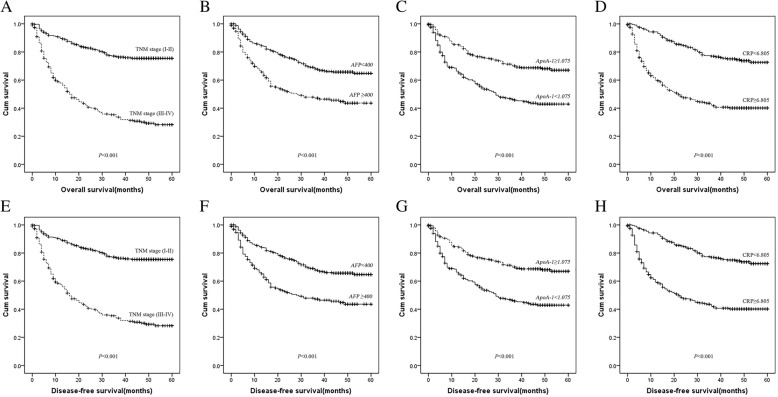
Analysis of OS and DFS in HCC patients by the Kaplan-Meier method. (**a**) OS in HCC patients by TNM stage; (**b**) OS in HCC patients by AFP level; (**c**) OS in HCC patients by ApoA-1 level; (**d**) OS in HCC patients by CRP level; (E) DFS in HCC patients by TNM stage; (**f**) DFS in HCC patients by AFP level; (**g**) DFS in HCC patients by ApoA-1 level; (**h**) DFS in HCC patients by CRP level

Kaplan-Meier survival curves were used to further explore the prognostic significance of independent predictors in HCC. In the whole cohort, the OS in the low ApoA-1 group was 24.78 ± 20.42 months, whereas it was 36.22 ± 19.36 months in the high ApoA-1 group (*P* < 0.001). The DFS in the low ApoA-1 group was 24.71 ± 20.35 months and was also shorter than that in the high ApoA-1 group (35.99 ± 19.42 months) (*P* < 0.001). Additionally, the OS in the low CRP group was 40.10 ± 16.85 months, whereas it was 22.63 ± 20.25 months in the high CRP group (*P* < 0.001). The DFS in the low CRP group was 39.85 ± 16.84 months, which was better than that in the high CRP group (22.57 ± 20.29 months) (*P* < 0.001) (Fig. 2).

### The novel AC score predicts survival time of HCC patients

**Fig. 3:**
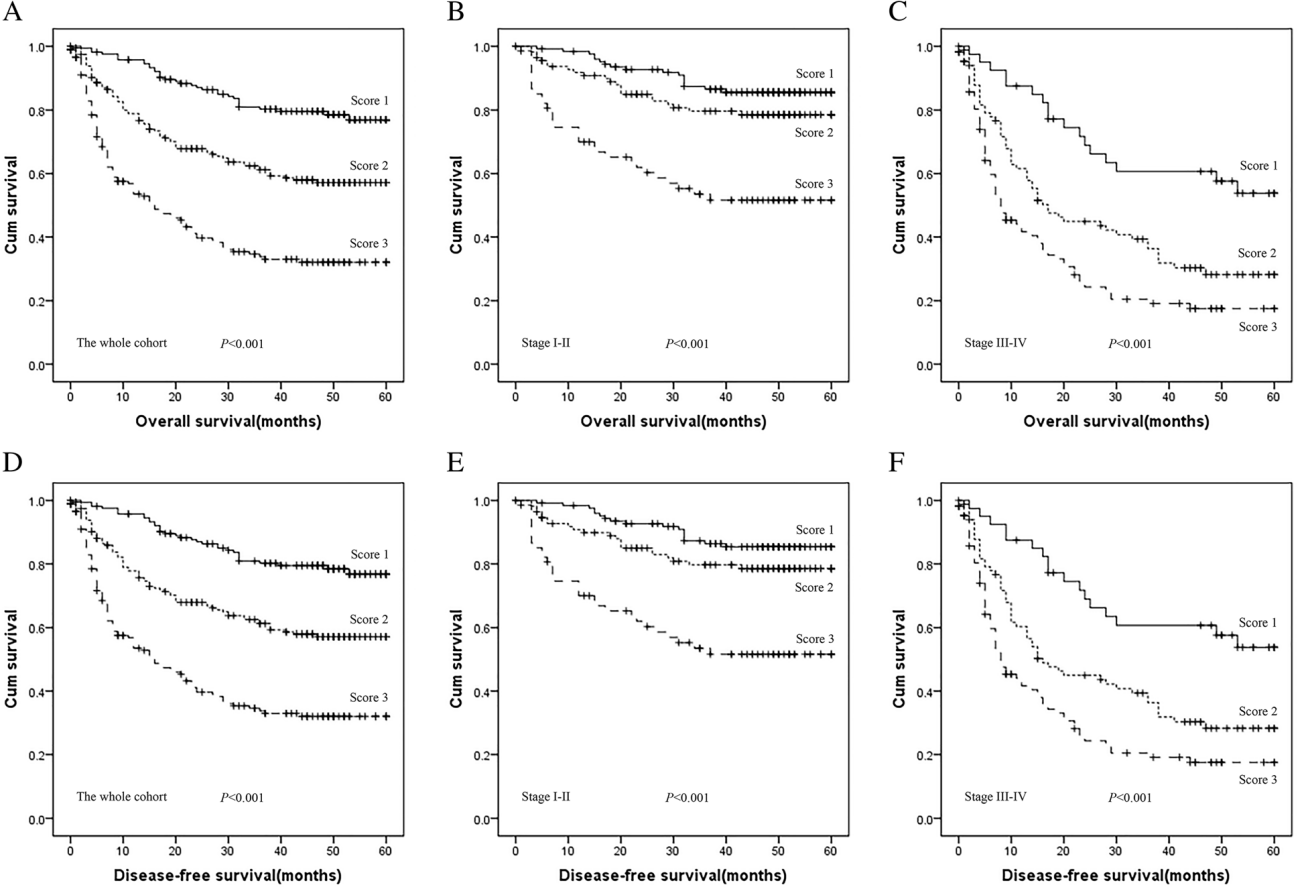
Prognostic significance of the AC score in HCC in the whole cohort and in those with different pathological disease stages by Kaplan-Meier survival curves. (**a**) OS of the whole cohort of patients with HCC; (**b**) OS of patients with early pathological stage (stage I-II) HCC; (**c**) OS of patients with advanced pathological stage (stage III-IV) HCC; (**d**) DFS of the whole cohort of patients with HCC; (**e**) DFS of patients with early pathological stage (stage I-II) HCC; (**f**) DFS of patients with advanced pathological stage (stage III-IV) HCC

**Fig. 4:**
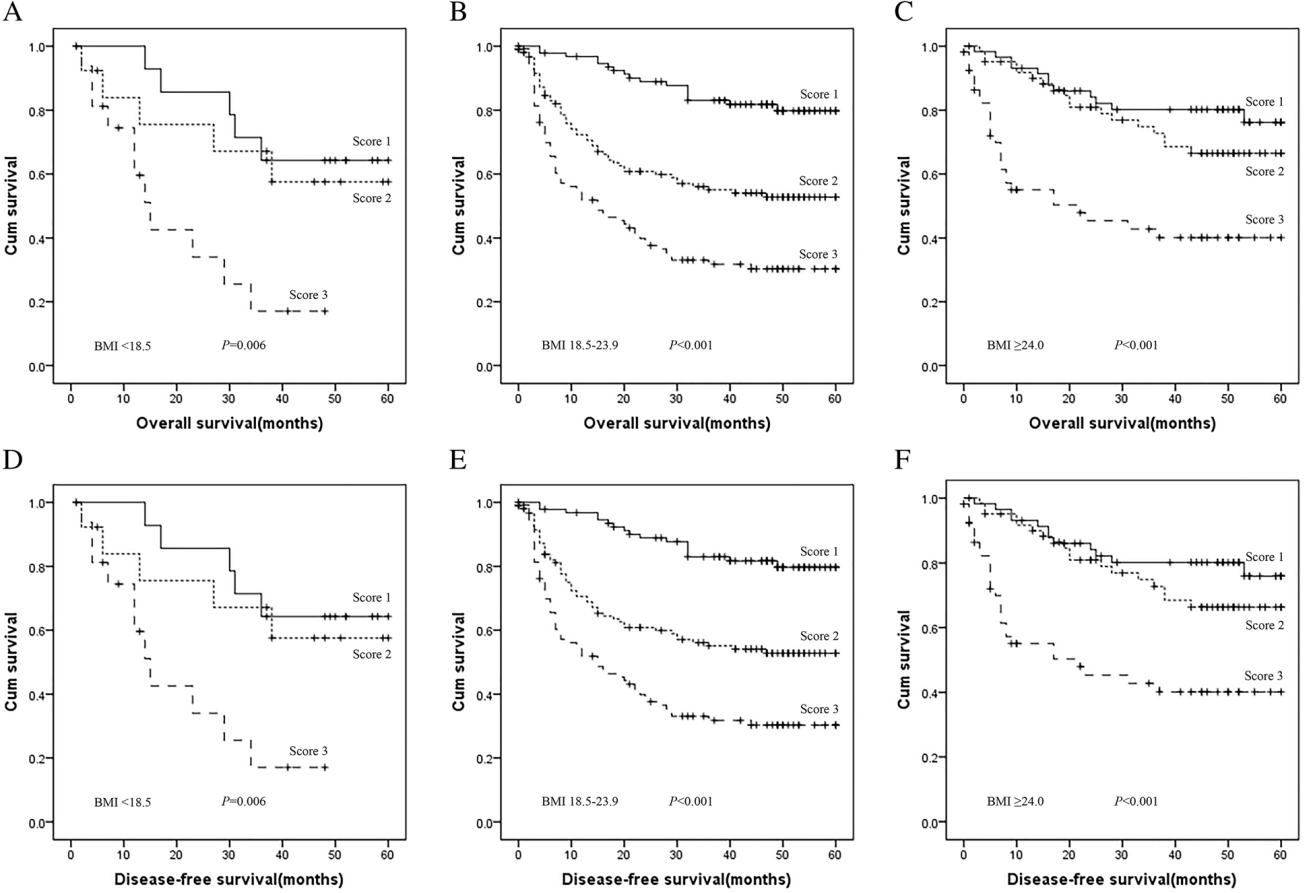
Prognostic significance of the AC score in HCC in different BMI groups according to Kaplan-Meier survival curves. (**a**) OS of HCC patients in the low BMI group (BMI < 18.5); (**b**) OS of HCC patients in the normal BMI group (BMI: 18.5–23.9); (**c**) OS of HCC patients in the high BMI group (BMI ≥24.0); (**d**) DFS of HCC patients in the low BMI group (BMI < 18.5); (**e**) DFS of HCC patients in the normal BMI group (BMI: 18.5–23.9); (**f**) DFS of HCC patients in the high BMI group (BMI ≥24.0)

As independent prognostic factors, ApoA-1 and CRP can be used to reflect the OS and DFS of HCC patients, which prompted us to develop a prognostic grouping system to better reflect the actual outcomes of patients with HCC. The relationships among ApoA-1, CRP and the clinical characteristics of HCC patients are summarized in Table 4. Specifically, the ApoA-1 and CRP levels have not been significantly correlated with differences in aetiology (HBsAg: *P =* 0.664, *P* = 0.780; HBeAg: *P =* 0.492, *P* = 0.754; alcohol behaviour: *P =* 0.802, *P* = 0.409; HCV Ab: *P =* 0.197, *P* = 0.075). Patients were allocated as follows: score of 1 (low risk: ApoA-1 ≥ 1.090 g/L and CRP < 3.00 mg/L), score of 2 (medium risk: ApoA-1 < 1.090 g/L or CRP ≥ 3.00 mg/L), score of 3 (high risk: ApoA-1 < 1.090 g/L and CRP ≥ 3.00 mg/L). Among the 539 patients, 166 (30.80%) HCC patients had an AC score of 1, 196 (36.36%) HCC patients had an AC score of 2, and 177 (32.84%) HCC patients had an AC score of 3. The OS of HCC patients differed significantly according to the AC score (score of 1: 41.78 ± 16.10 months; score of 2: 31.72 ± 20.07 months; score of 3: 19.18 ± 19.13 months, *P* < 0.001). The DFS of HCC patients also differed significantly according to the AC score (score of 1: 41.52 ± 16.15 months; score of 2: 31.54 ± 20.10 months; score of 3: 19.18 ± 19.12 months, *P* < 0.001). Furthermore, to explore the prognostic significance of the AC score in HCC patients, we analysed the prognostic effect in a selective subgroup stratified by TNM stage and BMI. Patients with a lower AC score had a significantly better OS compared with those patients with a high AC score; this was observed in patients with stage I-II or stage III-IV disease (*P* < 0.001, *P* < 0.001, respectively, for stages I-II and III-IV). Patients with a lower AC score had a significantly better DFS compared with those patients with a high AC score; this was observed in patients with stage I-II or stage III-IV disease (*P* < 0.001, *P* < 0.001, respectively, for stages I-II and III-IV), as shown in Fig. 3. Moreover, patients with a lower AC score had a significantly better OS and DFS in both the BMI ≥ 24.0 and BMI = 18.5–23.9 groups (Fig. 4).

**Table 4:** Main clinical characteristics of patients group according to ApoA1 and CRP levels

Variables	ApoA1(g/L)		CRP	
	Median (Range)	*p*-Value	Median(Range)	*p*-Value
Gender				
Male	1.10(0.15–1.80)	0.236	3.685(0.16–251.70)	0.083
Female	1.09(0.56–1.72)		2.60(0.10–92.96)	
Age (years)				
< 53	1.095(0.23–1.80)	0.539	4.08(0.16–210.65)	0.064
≥ 53	1.10(0.15–1.72)		2.89(0.10–251.70)	
TNM stage				
I-II	1.13(0.15–1.80)	< 0.001	2.255(0.10–241.15)	< 0.001
III-IV	1.03(0.16–1.64)		9.61(0.24–251.70)	
Tumor size(cm)				
< 5	1.13(0.15–1.80)	< 0.001	2.35(0.10–241.25)	< 0.001
≥ 5	1.03(0.16–1.64)		9.66(0.24–251.70)	
Tumor number				
Single	1.10(0.15–1.80)	0.544	2.73(0.10–251.70)	0.001
Multiple	1.10(0.41–1.64)		5.60(0.24–132.49)	
Node stage				
N0	1.10(0.15–1.80)	0.059	3.425(0.10–251.70)	0.005
N1–2	1.01(0.35–1.48)		8.03(0.57–194.10)	
Distant metastases				
No	1.10(0.15–1.80)	0.169	3.49(0.10–251.70)	0.008
Yes	1.04(0.16–1.56)		9.66(0.60–92.96)	
BMI (kg/m^2^)				
≥ 24.0	1.085(0.30–1.72)	0.756	2.89(0.16–241.25)	0.264
18.5–23.9	1.11(0.15–1.80)		4.03(0.10–251.70)	
< 18.5	1.08(0.41–1.77)		4.75(0.25–146.40)	
Alcohol behavior				
Never	1.10(0.15–1.80)	0.802	3.59(0.10–194.10)	0.409
Previous/Current	1.08(0.16–1.65)		3.56(0.16–251.70)	
Family history of cancer				
No	1.10(0.15–1.80)	0.772	3.56(0.10–251.70)	0.403
Yes	1.10(0.16–1.77)		3.765(0.30–126.24)	
HBs Ag				
Negative	1.10(0.15–1.80)	0.664	3.28(0.23–251.70)	0.780
Positive	1.10(0.15–1.80)		3.56(0.10–210.65)	
HBe Ag				
Negative	1.07(0.15–1.80)	0.492	3.71(0.16–251.70)	0.754
Positive	1.12(0.16–1.52)		3.79(0.25–130.04)	
HCV Ab				
Negative	1.10(0.15–1.80)	0.197	3.64(0.10–251.70)	0.075
Positive	1.02(0.26–1.64)		1.88(0.23–194.10)	
AFP (ng/mL)				
< 400	1.11(0.16–1.80)	0.020	3.10(0.10–251.70)	< 0.001
≥ 400	1.04(0.15–1.58)		8.20(0.30–241.25)

### The correlation between the AC score and the Clinicopathologic characteristics in HCC patients

The associations between the pretreatment AC score and clinicopathological variables in the 539 HCC patients are shown in Table 5. The AC score was associated with pathological stage, tumour size, tumour number, and node stage (*P* < 0.001, *P* < 0.001, *P* < 0.001, *P =* 0.005*,* respectively). However, no significant differences were observed in age, gender, distant metastases, BMI, alcohol behaviour, family history of cancer, or in the levels of AFP, HBsAg and HBeAg.

### Prognostic values of the individual serum AFP level, the AC score or their combinations in HCC patients

**Fig. 5:**
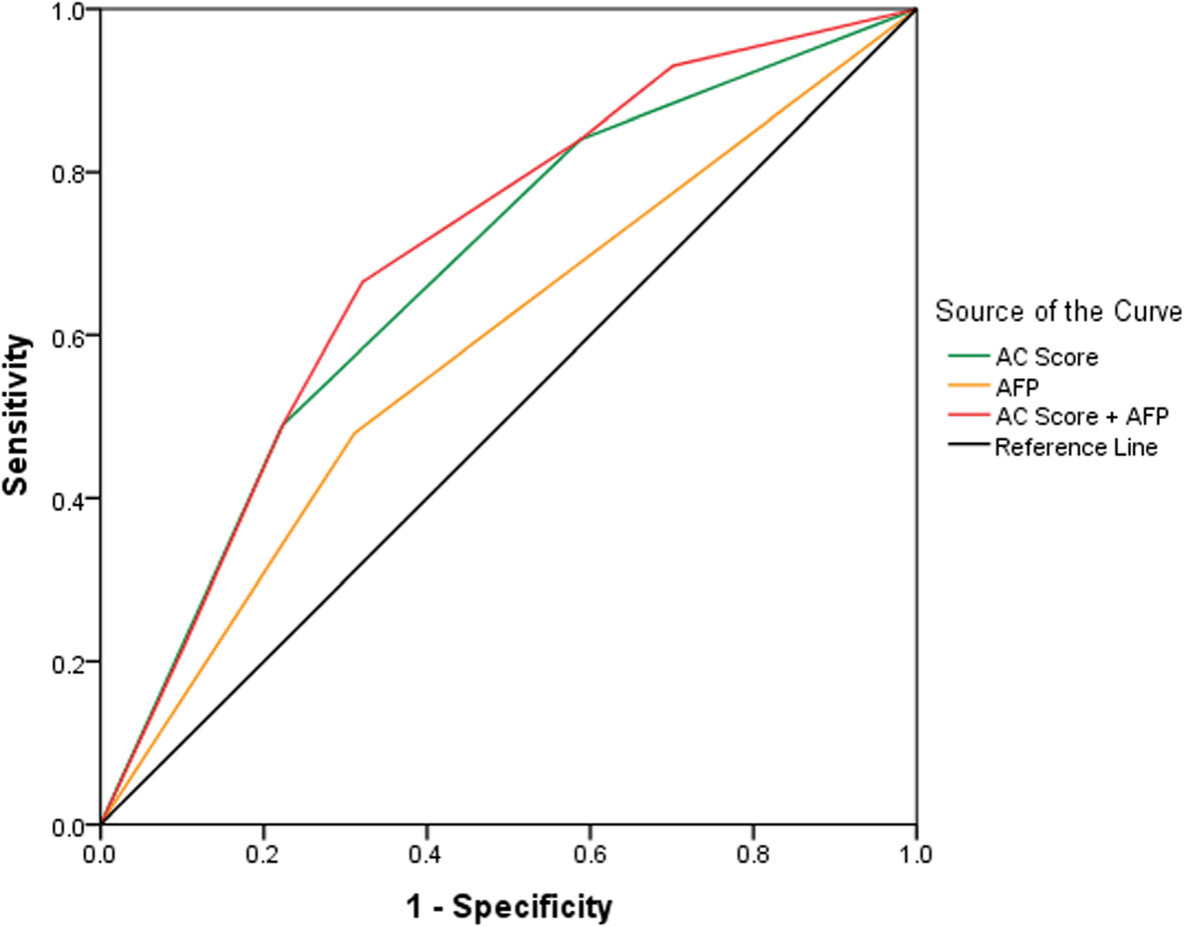
Comparison of the discriminatory ability of the AC score in HCC patients. The AUC of the AC score was higher than that of the AFP score alone (0.686 vs. 0.592), and the AUC of the combination of the AFP and AC scores (0.720) was superior to that of the AFP and AC scores alone

The ROC curve was plotted to assess the discrimination ability of the AFP level, the AC score, and the AFP-AC combination score in HCC prognosis, as shown in Fig. 5. According to the AUC, the prognostic value of the AC score was 0.676 (95% CI: 0.629–0.723, *P* < 0.001, sensitivity = 76.00%, specificity = 53.80%), which was higher than that of the AFP level (AUC: 0.584, 95% CI: 0.534–0.635, *P* < 0.001, sensitivity = 33.50%, specificity = 85.90%). To further improve accuracy, we combined the AFP and AC scores to establish models. The AUC of the combination of the AFP and AC scores (AUC: 0.700, 95% CI: 0.655–0.745, *P* < 0.001, sensitivity = 61.5%, specificity = 73.30%) was superior to that of the AFP and AC scores alone.

## Discussion

In our study, the prognostic values of biomarkers of lipid metabolism in HCC patients were evaluated. To our knowledge, this is the first large cohort study to evaluate the prognostic significance of the combination of ApoA-1 and CRP (AC score). We found that the levels of all the lipids assessed were significantly lower in HCC patients than those in the control group and that only the CRP level was higher in HCC than in controls. We found that an increased ApoA-1 level and a lower CRP level were significantly associated with poor survival. In addition, TNM stage and AFP level were independent prognostic factors for OS and DFS in HCC patients.

According to the multivariate analysis results, we combined the ApoA-1 and CRP levels to establish the AC scoring system and then divided the patients into three groups (score of 1, score of 2, and score of 3). We found that patients with a higher AC score showed more progressive disease and a poorer prognosis, not only in the entire cohort (For OS, *P* < 0.001; For DFS, *P* < 0.001) but also in the subgroups stratified by pathological stage (stage I-II and stage III-IV). Furthermore, we also used the AUC values to assess the discriminatory ability of the AC score in HCC and found that the AUC value of the AC score (AUC: 0.676, 95% CI: 0.629–0.723, *P* < 0.001) was higher than that of the AFP score alone. We also found that the AUC value of the combination of the AFP and AC scores (AUC: 0.700, 95% CI: 0.655–0.745, *P* < 0.001) was superior to that of the AFP and AC scores alone.

ApoA-1 has been reported to be a prognostic factor and is widely recognized and validated in several cancers, where it plays a role in anti-inflammatory [14], anti-apoptotic [15], and antioxidant activities [16]. In addition, inflammation and CRP levels are always elevated in malignancies [17]. Chronic inflammation, anti-inflammation, oxidative stress, lipids, and inflammatory cytokines have all been associated with tumorigenesis [18]. Therefore, we successfully defined a prognostic indicator, termed the AC score, by incorporating the above independent risk factors into a new system of patient stratification.

As the main protein component of high-density lipoprotein (HDL), ApoA-1 is synthesized predominantly in the liver and small intestine and is part of the apolipoprotein A1/A4/E family [19].

Through interaction with ATP-binding cassette transporters at the surface of cells in the periphery, which extract cholesterol and phospholipids, ApoA-1 initiates assembly of HDL particles; this, in turn, transfers fatty acids and ethanolamine back to cells for reuse and reverses the transport of cholesterol from tissue to the liver for excretion. ApoA-1 acts as a cofactor for lecithin-cholesterol acyltransferase (LCAT), which converts cholesterol to cholesteryl ester [20]. Many studies have focused on the relationship between ApoA-1 and tumours. Chang et al. [21] proposed that a high ApoA-1 level was associated with a decreased cancer risk, specifically with recurrence in breast cancer. Zhang et al. [22] indicated that patients with early-stage ovarian cancer have a reduced ApoA-1 level compared with normal individuals. In our previous study, we showed that a low ApoA-1 level was strongly correlated with poor OS and was an independent prognostic factor for survival in oesophageal squamous cell carcinoma [23], but the exact mechanism of ApoA-1 in carcinogenesis is unknown. Ma et al. [24] reported that ApoA-1 might be a promising therapeutic target to reduce recurrence and metastasis in HCC patients after resection. There are several explanations for why ApoA-1 might be a good therapeutic target. Firstly, ApoA-1 negatively influences the tumour microenvironment in terms of decreased overall metastasis and an increased accumulation of tumour-associated macrophages (TAMs) with an M1-like antitumour phenotype in direct contrast with the traditional anti-inflammatory and immunosuppressive functions [25]; ApoA-1 may also increase the level of tumour cell killing macrophages and may increase the recruitment of CD8 T cells and decrease the levels of the anti-apoptotic protein survivin within the tumour bed. Second, when injected or administered orally, ApoA-1 mimetic peptides could inhibit tumour growth and reduce the plasma levels of tumour-promoting lysophosphatidic acid (LPA) and oxidized lipids (which serve as potent tumour growth and angiogenic factors) in tumour-bearing mice [26]. Third, ApoA-1 influences the immune cell response to tumours via the modulation of cholesterol content in membrane lipid rafts [27]. Lastly, ApoA-1 could inhibit tumour cell proliferation through cell cycle arrest and could promote apoptosis in the regulation of the mitogen-activated protein kinase (MAPK) pathway [24]. Further research on ApoA-1 is needed to investigate its underlying mechanisms. Moreover, cancer-associated inflammation plays a key role in cancer initiation, progression, metastasis, and survival [28]. As an acute-phase protein, CRP is the prevailing marker of inflammation, and various studies have reported that CRP is increased in patients with tumours and that it is associated with a poor prognosis. CRP might play a direct role in the stimulation of inflammatory reactions in tumorigenesis, which is typically described by the GPS (Glasgow prognostic score) [29].

We determined that both ApoA-1 and CRP levels were independent prognostic variables for survival in patients with HCC. Hence, we suggested the prognostic scoring system for HCC, which involves the incorporation of CRP and ApoA-1; this system divides HCC patients into three risk groups. Moreover, the AC score and the AFP combination increased the AUC, which indicates the combined score can more accurately differentiate the prognosis of HCC patients. Our study had some limitations. Our study is a retrospective analysis of patients at our hospital, and thus the results need to be validated in large, multicentre prospective trials. The mechanism of ApoA-1 will be confirmed in a future study.

## Conclusion

Our study provides strong evidence to support that the AC score is a useful and more accurate predictive factor for OS and DFS in HCC, both in the entire cohort and in groups stratified by pathological stage. It would be significant to help clinicians identify high-risk patients out of all HCC patients. Our study had some limitations. Our study is a retrospective analysis of patients at our hospital, and the results need to be validated in large, multicentre prospective trials. The mechanism of ApoA-1 will be confirmed in a future study.

## Abbreviations

95% CI: 95% confidence interval
AFP: Alpha fetoprotein
ApoA-1: Apolipoprotein A-1
ApoB: Apolipoprotein B
BMI: Body mass index
CRP: C-Reactive protein
DFS: Disease-free survival
HCC: Hepatocellular carcinoma
HDL-C: High-density lipoprotein cholesterol
HR: Hazard ratio
LDL-C: Low-density lipoprotein cholesterol
OS: Overall survival
TC: Total cholesterol
TG: Triglyceride
